# Considering brain state for individualized functional connectivity-based rTMS

**DOI:** 10.1162/IMAG.a.1096

**Published:** 2026-01-12

**Authors:** Hallee Shearer, Jeffrey Eilbott, Fidel Vila-Rodriguez, Tamara Vanderwal

**Affiliations:** Department of Psychology, Northeastern University, Boston, MA, United States; BC Children’s Hospital Research Institute, Vancouver, BC, Canada; Department of Psychiatry, University of British Columbia, Vancouver, British Columbia, Canada; School of Biomedical Engineering, University of British Columbia, Vancouver, British Columbia, Canada

**Keywords:** brain state, precision psychiatry, transcranial magnetic stimulation, neurostimulation, naturalistic neuroimaging

## Abstract

Recent endeavors to optimize the efficacy of repetitive Transcranial Magnetic Stimulation (rTMS) treatment have focused on locating individualized stimulation targets using functional connectivity derived from functional Magnetic Resonance Imaging (fMRI) scans. Practically, this approach involves three main stages: target discovery, target localization, and treatment. As of now, each stage is typically conducted while participants are “at rest”, meaning they are not performing a task or being presented with a stimulus. While growing evidence suggests that the effects of TMS are sensitive to the state of the brain at the time of stimulation, brain state has largely been overlooked during the first two stages (target discovery and localization). Here, we consider the potential importance of brain state at each stage of individualized rTMS, reviewing the relevant (and interdisciplinary) literature, and providing some exploratory example cross-state analyses. We also explore how manipulating and constraining brain state with tasks or movie-watching may provide opportunities to improve the reliability of individualized rTMS targets.

## 1 Introduction

Repetitive Transcranial Magnetic Stimulation (rTMS) offers potential as a psychiatric treatment, especially for treatment-resistant depression (TRD) (Milev et al., 2016). Around 46% of individuals with TRD respond to rTMS and approximately 31% achieve remission (Fitzgerald et al., 2016). While encouraging, these numbers hold room for improvement. Recent optimization efforts have investigated the effects of personalizing treatment parameters such as stimulation intensity, pulse frequency, coil orientation, and target location to improve the overall efficacy of the treatment (Cash, Cocchi, et al., 2021; Klooster et al., 2022; Van Rooij et al., 2024). One notable effort is the recent FDA approval of SAINT, an accelerated rTMS protocol that utilizes personalized stimulation targets (Cole et al., 2020).

Previous research has explored the effect of the state of the brain at the time of stimulation. In the simplest sense, brain state is “what the brain is doing” at a given point in time. To study brain state, however, a more specific definition is needed. In line with previous work, here we will use the term brain state to describe the physiological state of the brain at a given point in time, with different brain states representing recurring patterns of neural activity (A. S. Greene et al., 2023). Greene et al. describe a brain state as the “product of a specified cognitive or physiological state” that is “characterized by a widely distributed pattern of activity or coupling” and “affects the future physiology and/or behavior of the organism.”

Research has shown that TMS pulses can have variable, even opposing, effects depending on the state of the brain at the time of stimulation (Bestmann et al., 2008; Janssens & Sack, 2021; Keil et al., 2014; Sack et al., 2023; Silvanto & Cattaneo, 2021). In light of this, some researchers are investigating ways to optimize brain state for TMS treatment (Bergmann, 2018; Caulfield & Brown, 2022; Sack et al., 2023; Schutter et al., 2023).

Another parameter that researchers have explored is localizating stimulation targets at the individual level with fMRI. Individualized FC-based target localization involves multiple steps—discovering and defining the optimal target, then locating the target for each individual, and then treatment. Brain state, as discussed above, has almost exclusively been considered for the final step. Should we consider brain state across the other stages of individualized target localization? In this brief review, we will consider brain state, and the potential of using acquisition and treatment conditions (i.e., movie-watching or tasks) to manipulate brain state, at each stage of the individualized rTMS process. Since this concept lies at the intersection of typically separate fields, we begin by reviewing relevant literature within each field.

## 2 Brain State during TMS

A 2008 review by Silvanto & Pascual-Leone showed that the neural effects of TMS stimulation depend on brain state, and mounting evidence since then has further supported this relationship (Bestmann et al., 2008; Janssens & Sack, 2021; Keil et al., 2014; Sack et al., 2023; Silvanto & Cattaneo, 2021; Silvanto & Pascual-Leone, 2008). In humans, an early study stimulated the visual cortex with and without an adaptation period prior to stimulation (Silvanto et al., 2007). During the adaptation period, the participant was shown a certain color to evoke adaptation (decrease the excitability) in the neurons that are tuned to fire in response to that color. Without prior adaptation, a TMS pulse to the visual cortex induced a colorless spot of light in the visual field called a phosphene (i.e., TMS after no color cue led to a colorless phosphene). After adaptation, however, a TMS pulse to the same area of cortex excited the adapted neurons preferentially, in turn inducing a phosphene *of the adapted color* (i.e., TMS after green adaptation led to a green phosphene). Since adaptation leads to lower excitability, the authors suggested that TMS may preferentially affect less excitable neurons and is therefore state-dependent in some significant ways.

This idea was explored in more detail by Silvanto and Cattaneo (Silvanto & Cattaneo, 2021) by including the variable of stimulation intensity. In this study, participants were primed with stimuli of a particular color and line orientation, then were presented with a test stimulus that was either incongruent, partially congruent (either color or orientation was congruent), or fully congruent to the priming stimulus. When presented with the test stimulus, TMS was administered at either 60% or 120% of the intensity required to induce a visual phosphene, to either the visual cortex or the vertex (control condition). Participants were then asked to report the color of the test stimulus, and response latency was measured. High-intensity TMS to the visual cortex impaired response time of fully and partially congruent correct trials, while low-intensity TMS improved performance of fully incongruent correct trials, relative to TMS of the vertex (control). Thus, by varying the stimulation intensity, the ability of a TMS pulse to either excite or inhibit was dependent upon current brain activity, cortical excitability, and stimulation intensity. These findings highlight the complex network of interactions involved in the brain state dependency of TMS, and show that TMS stimulation likely depends on underlying brain activity in more elaborate ways than previously thought.

Perhaps even more surprising, multiple studies have shown that manipulating brain state during TMS treatment in clinical populations causes significant differences in treatment outcomes. Fitzgerald et al. (2008) primed the right DLPFC with 6 Hz rTMS stimulation prior to 1 Hz rTMS treatment. The priming led to a greater reduction in depressive symptoms than sham priming (30% reduction in MADRS scored in the priming group; 13.2% reduction in the sham group after 4 weeks, p < 0.05, N = 60). In another study, patients with depression were instructed to focus on either positive emotions (N = 17), negative emotions (N = 15), or neither (N = 25) during TMS treatments of the prefrontal cortex using an H-coil (thought to induce deeper stimulation). The treatment was less effective in the group that focused on negative emotions compared to the group that focused on neither (mean improvement in Hamilton Depression Rating Scale 24 of 9.7 and 15.8 in the negative and neither groups, respectively; mean improvement in Beck Depression Inventory – II of 4.2 and 10.0 in the negative and neither groups, respectively) (Isserles et al., 2011). Another study of adults with depression attempted to cognitively manipulate activity in the rostral anterior cingulate cortex prior to treatment by having participants perform a computerized cognitive task involving sustained attention and working memory. They found improved response and remission rates from 10 Hz rTMS of the left DLFPC relative to sham priming, with 42% of patients achieving remission in the active priming and rTMS group and 17% in the sham priming (and active rTMS) group (Li et al., 2016). Although not a clinical sample, another behavioral/cognitive manipulation study showed that administration of rTMS to the DLPFC 10 min after the reactivation of a fear memory decreased physiological expression of fear (measured via skin conductance response) compared to controls that received rTMS without the reactivation of the fear memory (Borgomaneri et al., 2020). A recent preprint reported that eliciting a calm state with a nature video during intermittent Theta Burst Stimulation (iTBS) improved PTSD avoidance scores (from the PTSD checklist for DSM-5) more than eliciting a cognitively demanding state with an N-back task during the treatment (Oathes et al., 2024). Interestingly, this finding was only present when the treatment was administered using individualized targets, and not when using standard group-level targets. Together, these investigations illustrate the importance of considering brain state for rTMS treatment administration, but none of them considered brain state in relation to target localization, and only Oathes et al. (2024) considered treatment that uses individualized fMRI-based targets.

Recent work by van Rooij et al. (2024) called for a nuanced approach when attempting to control brain state for TMS. While it is, indeed, difficult to manipulate brain state directly and precisely, the choice of acquisition or treatment condition offers an important—and feasible—method of better constraining brain state. In other words, by carefully fine tuning the sensory inputs, we may increase the probability of modulating constrained brain states in participants.

## 3 Considering Brain State for Individualized rTMS

**Fig. 1.**
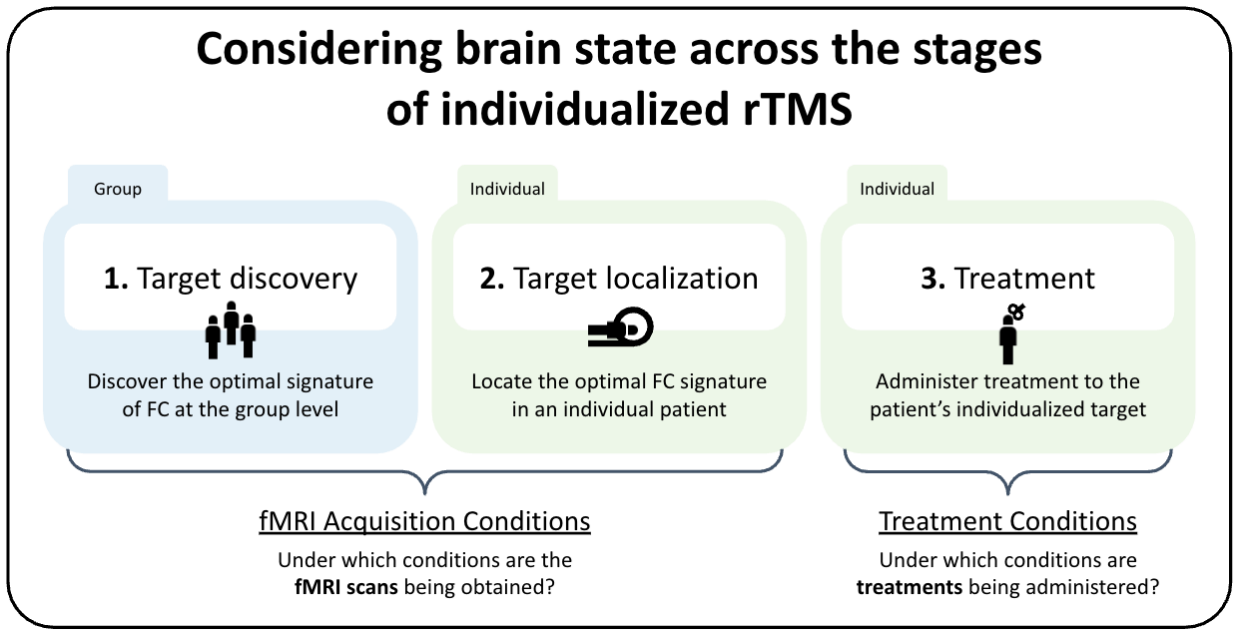
The three stages of individualized rTMS treatment. First, the FC signature of the optimal rTMS target is explored and defined at the group level (1). Then, that FC signature is localized in an individual patient (2). The first two stages involve fMRI scans with acquisition conditions that could be carefully selected. Lastly, rTMS treatment is administered to the patient at the previously localized target under certain treatment conditions (3).

A main focus of recent endeavors to optimize rTMS efficacy has been on the individualization of stimulation locations, often determined with individual fMRI scans (Cash, Cocchi, et al., 2021; Cash, Weigand, et al., 2021; Fox et al., 2013; Luber et al., 2017). This “precision medicine” approach, referred to as individualized rTMS, requires three stages: target discovery, target localization, and treatment (Fig. 1). As of now, the acquisition/treatment condition for all three stages has mainly been resting state. The impact of brain state on the former two stages has not (to our knowledge) been investigated, and neither has the impact of consistency—or lack thereof—of brain state across these stages.

### 3.1 Target discovery for a given population

Target discovery often involves investigating the relationship between functional connectivity patterns and treatment response to identify an optimal functional connectivity pattern that may be used as an individualized target (Cash, Cocchi, et al., 2021; Cash, Weigand, et al., 2021; Fox et al., 2012, 2013). For example, Fox et al. (2012) found retrospectively that more effective rTMS targets in the DLPFC were more anticorrelated with the subgenual cingulate cortex, and therefore proposed an optimal individualized rTMS target as the part of the DLPFC most anticorrelated with the subgenual. However, this study was limited by a relatively small sample size (n = 27) —a common limitation in TMS research. A recent study with a larger, more naturalistic sample that did not exclude psychiatric comorbidities, only observed this relationship when the sample was restricted to meet the strict inclusion criteria of the previous study or when clinical covariates were included in the analysis (Khosravani et al., 2025). This suggests that the DLPFC-SGC individualized TMS target may only be effective in a specific subset of patients. As described by Schmaal (2023), it is also possible that similar symptoms (e.g., melancholy or anhedonia) are driven by different circuits in different patients. This might mean that the process of target discovery itself may need to become increasingly individualized, perhaps with different targets for different subtypes.

**Fig. 2.**
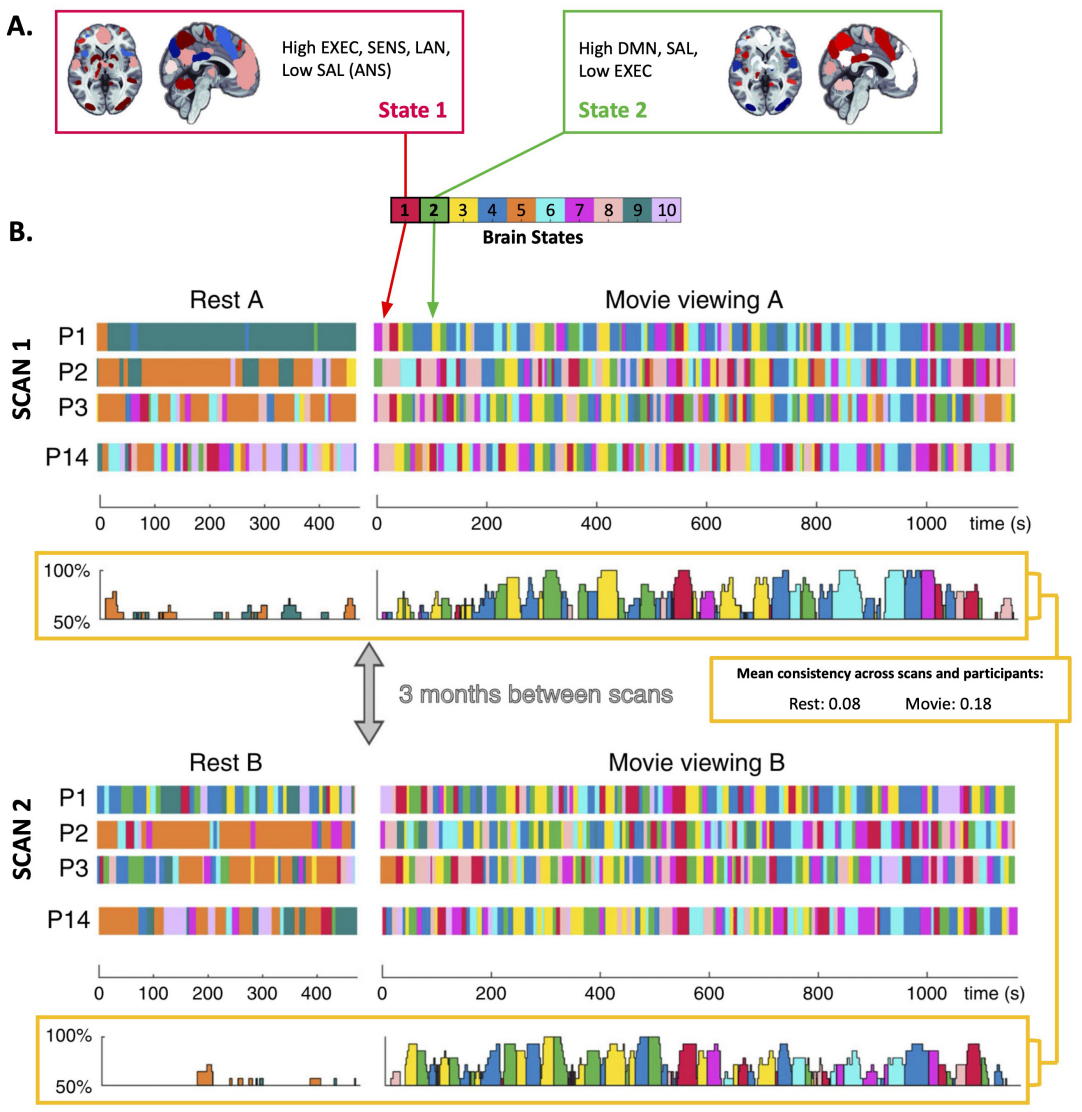
Brain states during rest and movie-watching across repeated scans. Adapted from van der Meer et al. (2020). Brain state is more consistent across participants (denoted by P, e.g., P1 is participant 1) and scans during movie watching than resting state. (A) The first two of ten brain states estimated with a Hidden Markov Model are shown in A to demonstrate the characterization of brain state as a configuration of network activity that recurs throughout the scan. State 1 (red) is an executive network-based state, and state 2 (green) is a state with strong default network contribution. (B) The occurrence of these ten brain states throughout a resting-state run and a movie-watching run for four participants (out of 14 total) on 2 days 3 months apart. At the bottom of each scan, the temporal consistency across participants is shown between 50–100%, where 50% represents a conservative estimate of chance for resting state, which mainly involves states 5 and 9. Mean consistency (Jaccard overlap index) of brain state expression across scans and participants is higher with movie-watching (0.18) compared to rest (0.08).

All of these foundational target localization studies have been conducted using “task free” resting-state fMRI. Though the configurations and network organization that occurs during a resting-state scan is fairly reliable and similar across participants (Damoiseaux et al., 2006; Pannunzi et al., 2017; Shehzad et al., 2009), variability exists, and in particular, the dynamics or timing of a given brain state occurring are highly variable across participants (van der Meer et al., 2020) (Fig. 2). More constrained acquisition conditions with time-locked stimuli may be advantageous to promote consistency both within and across subjects (i.e., synchronization). This could improve our ability to detect meaningful differences between subjects associated with the brain-behavior relationship of interest. This is a wide-open question that would require a major effort (including collecting new data with new acquisition states such as tasks or movie-watching in a fairly large sample) to answer.

### 3.2 Target localization in an individual patient

At this stage, the pattern of FC identified as the “optimal” rTMS target from larger studies is located within a given patient based on their individual fMRI scan. Following the previous example, this might involve locating an individual subject’s subregion within the DLPFC that is most anticorrelated with the subgenual cingulate cortex. Since this is done at the individual subject level, data quality is of the utmost importance, and long scans are often required to locate such a pattern. Recent efforts to address data quality include analytical approaches (Cash, Cocchi, et al., 2021) and the use of multi-echo fMRI acquisition parameters (Lynch et al., 2020), but acquisition condition has hardly been considered.

The process of target localization is likely sensitive to brain state. The location of the part of the DLPFC that is most anticorrelated with the SGC during rest may be different than the location of that pattern during, for example, an anxiety-inducing task, or when viewing a sad scene from a film. While we cannot perfectly induce a certain brain state, we can increase the likelihood of evoking certain brain states by carefully choosing the acquisition condition for the scan. By maximizing the duration of the scan spent in a particular brain state of interest, and collapsing across time during the functional connectivity analysis, it is possible that that such data would yield a more reproducible individualized target localization. Further, if the known target is associated with specific epochs of the scan (i.e., epochs that reliably induce a certain brain state, psychological state, or physiological state relevant to symptoms), those epochs could potentially be leveraged with a quasi-dynamic analysis.

Theoretically, it would be ideal to localize the target during the same brain state, or at least acquisition condition, as was occurring when the target was defined. It is unlikely that a pattern of interest identified under condition A would translate to the same pattern of interest under condition B. We explored this idea in our exploratory analysis by comparing the localization of SGC-DLPFC targets (originally defined during resting state), between resting state and movie watching acquisition conditions. Our results suggest that (perhaps unsurprisingly) the location of the target depends on the acquisition condition of the localization scan. Therefore, consistency in acquisition conditions between target discovery and target localization scans may prove advantageous.

### 3.3 Treatment

The final stage of individualized rTMS is the treatment stage, where participants receive rTMS stimulation to the previously identified individualized target that may be precisely located with neuronavigation technology and e-field modeling.

The lack of constraint of brain state during the treatment phase seems to introduce variability in response. As previously discussed, manipulation of brain state during TMS stimulation can influence outcomes (Fitzgerald et al., 2008; Isserles et al., 2011; Li et al., 2016; Oathes et al., 2024). It may, thus, be important to constrain brain state, likely through careful selection of treatment conditions, for the duration of each rTMS treatment as well as across repeated treatments.

Certain brain states may be more beneficial than others for rTMS treatment administration, creating an opportunity for TMS stimulation to function synergistically with brain state (Schiena et al., 2020). This idea has been explored by work administering TMS during therapy-based interventions (Donse et al., 2018; Neacsiu et al., 2018). One area where brain state has been considered and implemented already is in psychotherapeutic interventions. For example, exposure therapy relies on the activation of fear-related states (Foa & McLean, 2016), and imagery rescripting involves reactivating traumatic memories (Arntz, 2012). However, further research is necessary to unravel the likely complex interactions between specific brain states, acquisition/treatment conditions, disorders, and targets. For example, a brain state induced by an emotional regulation task may prove beneficial for the stimulation of an SGC-DLPFC target for treating depression, or a corticothalamic-dominant brain state induced by suspenseful movie clips may be beneficial for the stimulation of a limbic-accessing target for treating anxiety.

Finally, we can consider whether rTMS treatments should be administered under the same conditions as the discovery and localization scans. While this question cannot be answered with existing data, we hypothesize that consistency across all three conditions would be advantageous, and further, that using a more constrained condition that decreases variability within and between participants would help. With this consistency, we could be more confident that the pattern of FC identified as the optimal target would be stimulated with the treatment. However, this assumes that the mechanism underlying the stimulation of the optimal target requires stimulation of the pattern in that given state. It is also possible that the optimal treatment state differs from the optimal discovery and localization states, in which case efficacy would outweigh the benefits of consistency, and one state would be used for steps 1 and 2 above, and another for step 3.

### 3.4 Alternative acquisition conditions for individualized rTMS

Two acquisition conditions will be discussed here that have been shown to evoke more systematic patterns (especially with regard to timing during a scan): conventional tasks like an emotion identification task or n-back task, and movie-watching (A. S. Greene et al., 2023). Tasks provide the ability to infer brain states through the relationship between task events and brain connectivity (Shine et al., 2016) with certain task-induced states offering improved prediction of individual-level traits (D. J. Greene et al., 2020). These brain states are reliable enough to be decoded with FC data (Shirer et al., 2012). Tasks also appear to constrain the number of dominant brain states and increase the amount of time spent in these states (Chen et al., 2015). Further research is required to determine whether tasks can induce brain states that are favorable specifically for individualized rTMS.

Movie-watching as an acquisition state may offer some relevant advantages over both traditional tasks and resting state (Eickhoff et al., 2020; Finn, 2021; Nastase et al., 2020; Sonkusare et al., 2019; Vanderwal et al., 2019). In a recent study, 14 participants watched the same movie and underwent task-free resting-state scans during two separate fMRI scans, with a 3-month interscan interval. Brain states were identified with a Hidden Markov Model, both across participants and scans as illustrated in Figure 2 (adapted with permission from van der Meer et al., 2020). They found significantly greater mean consistency across participants and repeat scans with movie-watching relative to rest (assessed with the Jaccard overlap index, 0.18 for movies and 0.08 for rest averaged across the scan, and with moments of higher state consistency occurring during specific timepoints in the movie). These results suggest that movie-watching induces more reliable brain states both across subjects and across repeated scans, and that the “order of appearance” of those brain states is, in some instances, systematic and time-locked to the stimulus. As such, it seems that movie-watching could be leveraged to improve consistency of brain states across the three stages of individualized rTMS.

Movie-watching can be particularly advantageous for scanning clinical populations (Eickhoff et al., 2020). For example, movie-watching can improve scan compliance by decreasing both head motion and sleep in the scanner (Frew et al., 2022; Wang, Han, et al., 2017), both of which remain major, ongoing concerns when scanning patients. Specifically for individuals who move their head more in the scanner, movie-watching has been shown to reduce the linear increase in head motion throughout the duration of the scan (Frew et al., 2022). This could enable longer scans during which head motion would otherwise be prohibitive. Movie-watching can also improve the multivariate test-retest reliability of functional connectivity estimates (Wang, Ren, et al., 2017), especially in visual and temporal regions (Shearer et al., 2024).

Movies are also uniquely suitable to elicit brain activity of interest in a naturalistic manner. For example, emotional movie clips have been used to study FC of adolescents with depression (Gruskin et al., 2020), and movie clips of hair-pulling have been used to study FC associated with trichotillomania (Lee et al., 2010), Alice in Wonderland has been used to study BOLD-signal responses in psychosis (Rikandi et al., 2017), and movie clips with varying levels of background noise and conversations have been used to study ADHD (Salmi et al., 2020).

**Fig. 3.**
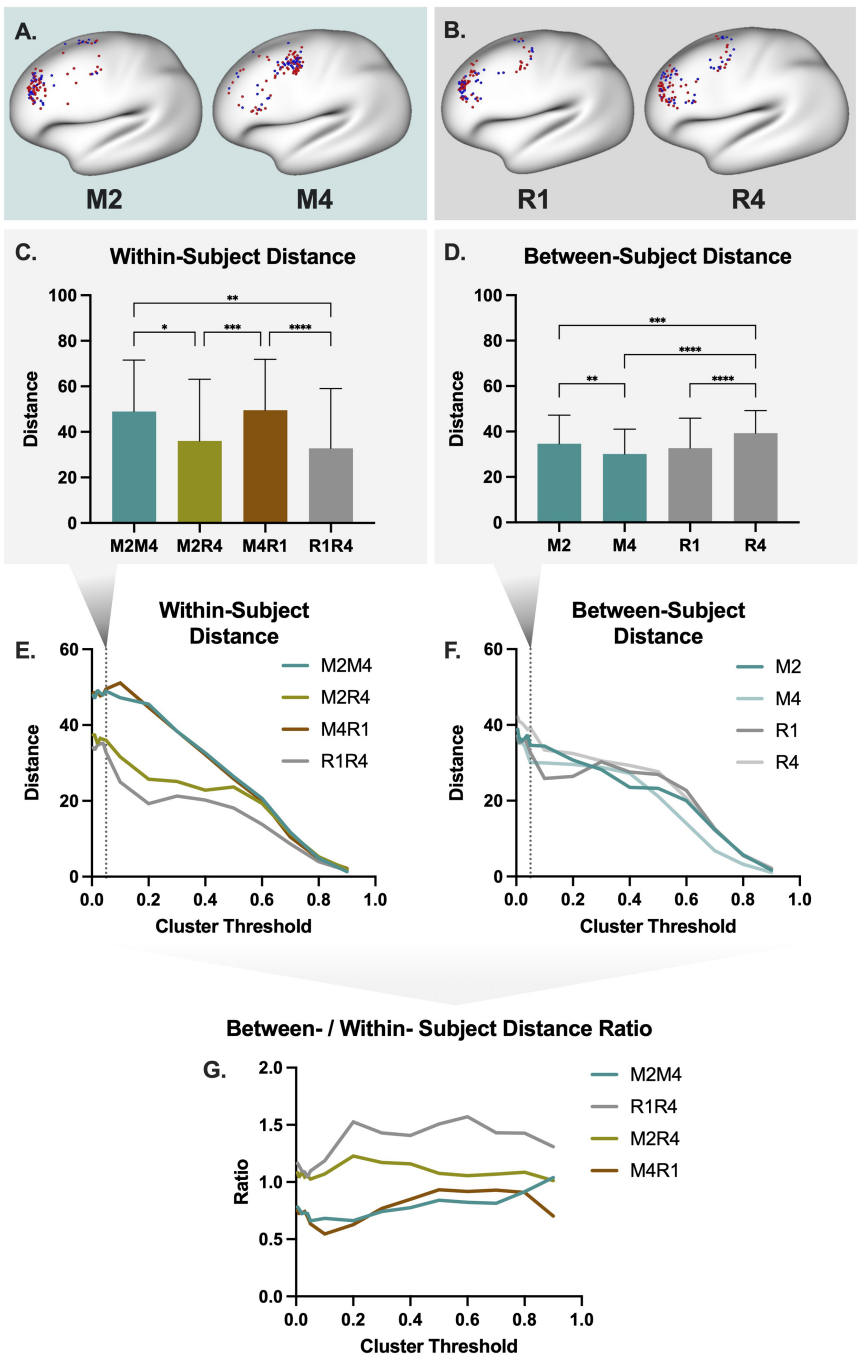
Localization of resting-state FC defined rTMS targets with rest and movie-watching FC. We localized individualized rTMS targets in a healthy adult sample (n=108) following the methods from Cash, Cocchi, et al. (2021). Two resting-state scans and two movie-watching scans (different movie clips in each scan) from the Human Connectome Project (HCP) 7 Tesla release were used. Both within-subject (A) and between-subject (B) distances between targets for each scan were computed and compared across movie and rest runs (see Supplementary Materials for more details). The locations (A-B), reliability (C, E), and variation between subjects (D, F) of individualized targets varied with acquisition conditions. Between-/within-subject distance of targets, of which a high value is optimal, also appears dependent upon condition (G). This exploratory initial investigation in healthy adults suggests that consistency in acquisition conditions affects the localization of individualized rTMS targets, but further investigation is required. It is important to note that these data may underestimate the utility of movie-watching data as M2 and M4 contain clips of different movies and thus are not ideal test-retest conditions. M2 = Movie 2, M4 = Movie 4, R1 = Rest 1, R4 = Rest 4. Targets are colored by sex in (A) and (B) (red = female, blue = male). Results in (C) and (D) are shown at a cluster threshold of 5% with significance level of *p ≤ 0.05, **p ≤ 0.01, ***p ≤ 0.001, and ***p ≤ 0.0001. Distance measures are all geodesic, in millimeters.

Any shift from resting state to movie-watching (or task) for individualized rTMS would have significant and costly implications. It appears unlikely that a specific FC target pattern (e.g. the region of the DLPFC most anti-correlated with the subgenual cingulate) would occur at the same anatomical location from movie to rest; however, this question has not yet been addressed in the literature. An exploratory demonstration into this question with actual data is shown in Figure 3.

In the exploratory demonstration, we located individualized targets with both resting-state and movie-watching FC data in healthy adults. Importantly, the nature of these individualized targets was discovered with resting-state data (Fox et al., 2012). Thus, the targets located with resting-state FC represent congruent acquisition conditions (i.e., rest-rest) between stage 1 and stage 2 of the individualized rTMS process. The targets located with movie-watching FC represent incongruent acquisition conditions (discovered in rest, localized in movie data). The results suggest that consistency in fMRI acquisition conditions affects individualized target localization. Though this may seem obvious, multiple studies have shown that much of the intrinsic organization of the functional connectome persists in both movie and resting state (Demirtaş et al., 2019; Kim et al., 2018; Vanderwal et al., 2017), so the fact that target localization changes across states here is a valuable starting point for future studies, and many levels of further investigation are necessary.

It is likely that movie-based targets would need to be defined using movie-fMRI data based on clinical outcomes (just as was done with resting state), and the validity of those new movie-targets would then need to be tested. This would be a major endeavor for the field and would require the addition of a movie scan to ongoing TMS projects. We suggest that the advantages of movie-fMRI may warrant the costs and efforts of redefining personalized rTMS targets under conditions other than task-free rest. Given the current lack of replication of the leading candidate target for rTMS in depression, efforts may need to start again from the target discovery stage, taking into account what has been learned from these important pioneering efforts. These new efforts are likely to focus more on discovering different targets for different subtypes (Siddiqi & Fox, 2024), and it may also be timely to consider the effects of acquisition condition at the discovery stage.

## 4 Conclusion

The optimization of brain state for individualized rTMS could offer various advantages. Both traditional tasks and movie-watching as acquisition conditions provide ways to constrain brain state. Movie-watching offers the added advantage of ecological validity or potential clinical relevance of the evoked brain states. This technique could be used to evoke brain states of interest, and to reliably reproduce potential “optimal” brain states. While the optimal brain states for target discovery, localization, and treatment are currently not known, the sensitivity of TMS stimulation to brain state warrants further exploration of this idea. A concurrent TMS-fMRI study of the effects of TMS pulses during various movie-watching brain states could provide valuable data for this purpose. In the meantime, we suggest that the reproducibility of brain states and the goodness-of-fit between movie-fMRI and clinical populations warrant the consideration and discussion of movie-watching as an acquisition condition for the three stages of individualized rTMS.

## Supplementary Material

Supplementary Material

## Data Availability

Original data used in this study are available at the HCP website (https://db.humanconnectome.org/). Code used to perform these analyses is available at our Github repository: https://github.com/halleeshearer/brain_state_tms.
