# Supplementary material for: Considering brain state for individualized functional connectivity-based rTMS
Source: Imaging Neurosci (Camb). 2026 Jan 12;4:IMAG.a.1096. doi: 10.1162/IMAG.a.1096 (PMC12797142; doi:10.1162/IMAG.a.1096)
Supplement: Supplementary Material [file IMAG.a.1096_supp.pdf]

Supplementary Methods

Data

All data were from the HCP 7T release (Van Essen et al., 2013). Data from a total of 184 healthy adult participants were collected during rest and while watching movie clips at the University of Minnesota with a Siemens Magnetom scanner (TR = 1000 ms, see https://www.humanconnectome.org/hcp-protocols-ya-7t-imaging for parameters). Resting state was eyes-open with a bright fixation cross on a dark background. Movie stimuli contained three shorter movie clips (3:48 to 4:19 minutes long) at the end. Four movie runs and four rest runs were available. We selected only the two Movie runs that contained Hollywood movie clips (as opposed to Creative Commons clips). We then selected the two Rest runs with phase encoding that matched the Hollywood movie runs. Therefore, we used Rest1, Rest4, Movie2, and Movie4 (each 15-16 minutes long). Rest1 and Movie2 (with posterior-anterior phase encoding) were collected on the first day, and Rest4 and Movie4 (with anterior-posterior phase encoding) on the second day. Written informed consent was obtained from each subject by the HCP committee and approved by the Washington University institutional Review Board (Van Essen et al., 2013).

Participants

Participants were healthy young adults between the ages of 22 and 36 years old. This dataset included sets of mono- and dizygotic twins, as well as siblings. After excluding 73 subjects with mean framewise displacement greater than 0.2 mm in any functional run, and two subjects based on quality control checks (unusually low correlation between Rest1 and Rest4 FC

matrices), our sample included 109 subjects with a mean age of 29.4 ± 3.4 years and 64 females (24 sets of monozygotic twins, 14 sets of dizygotic twins, four siblings, 29 unrelated).

Processing

HCP minimally pre-processed data were used(Glasser et al., 2013), for which no slice time correction was performed, spatial processing was applied, motion was corrected with FLIRT-based motion correction (no motion censoring was performed, subjects with mean FD > 0.2 mm were excluded), and structured artifacts were removed using ICA + FIX (independent component analysis followed by FMRIB's ICA-based X-noisifier). Data were represented as a timeseries of grayordinates in CIFTI format (i.e., cortical surface vertices and subcortical standard-space voxels). The first 30 TRs of each scan were removed, as well as 20-second epochs, and 10 TRs after each epoch, of rest that occurred between clips in the movie scans. We also removed temporally corresponding epochs from rest scans (20 seconds and the 10 TRs after each epoch). The runs were cropped to match the length of runs within each day. The resulting Rest1 and Movie2 scans were 704 TRs in length, and Rest4 and Movie4 were 687 TRs in length. The mean framewise displacement (FD) for these runs was 0.11 mm for Rest1, 0.11 mm for Movie2, 0.14 mm for Rest4, and 0.12 mm for Movie4. Of note, the differences in FD from Rest1 to Rest4 (0.023) and from Movie2 to Movie4 (0.017) were not significantly different ($p = 0.12$).

Functional connectivity

The subgenual cingulate cortex (SGC) was defined as the Glasser parcel number 164, and the left DLPFC was defined according to Cash et al., 2021. ROI masks were created with Connectome Workbench (Marcus et al., 2013).

Seedmap analysis

For each scan (Rest1, Movie2, Rest4, Movie4), group-average SGC connectivity maps were created by first calculating each individual's connectivity map between the average of the SGC ROI timecourse, and the rest of the brain's surface vertices, then averaging across these maps at the group level. The DLPFC ROI was removed from this group-average map. The remaining seedmap vertices were averaged to obtain a single timecourse that represents a proxy for the group-level subgenual timecourse for each scan. This proxy timecourse was then regressed onto each individual's DLPFC BOLD data to obtain each individual's SGC-DLPFC seedmap per condition.

Individualized target localization

These seedmaps were then thresholded to keep the top 5% of anti-correlated vertices. The largest cluster of contiguous vertices was selected for each map and the center of gravity of the cluster was identified as the vertex with the smallest sum of distances to all other vertices in the cluster. This center of gravity was identified as the target. This resulted in an individualized target for each subject and scan. This analysis was also repeated at a range of cluster thresholds from 0.5% to 95%.

Within-subject distances

Within-subject distances between individualized targets were calculated for each pair of cross-day scans, and each cluster threshold, with the workbench command -surface-geodesic-distance function. To improve computation speed, a distance matrix was first computed with the distances between all unique pairs of DLPFC vertices. This distance matrix was then indexed to find the distance between pairs of individualized targets. Distances (from the 5% cluster threshold targets) were compared statistically with the Friedman test and Dunn's post test using GraphPad Prism version 10 for Mac (GraphPad Software, Boston, Massachusetts USA, www.graphpad.com).

Between-subject distances

For each subject, condition, and cluster threshold, the distance between the subject and every other subject was calculated and averaged. To obtain a measure of between-subject distance per scan, these distances were further averaged across subjects. Distances (from the 5% cluster threshold targets) were compared statistically with the Friedman test and Dunn's post test using GraphPad Prism version 10 for Mac (GraphPad Software, Boston, Massachusetts USA, www.graphpad.com).

Ratio of between- to within-subject distance

For each scan and cluster threshold, the average between-subject distance was divided by the average within-subject distance to obtain a ratio influenced by both the reliability, as well as the individual variation, of the individualized targets.

References

Cash, R. F. H., Cocchi, L., Lv, J., Wu, Y., Fitzgerald, P. B., & Zalesky, A. (2021). Personalized connectivity-guided DLPFC-TMS for depression: Advancing computational feasibility, precision and reproducibility. *Human Brain Mapping*, *42*(13), 4155–4172. https://doi.org/10.1002/hbm.25330

Glasser, M. F., Sotiropoulos, S. N., Wilson, J. A., Coalson, T. S., Fischl, B., Andersson, J. L., Xu, J., Jbabdi, S., Webster, M., Polimeni, J. R., Van Essen, D. C., & Jenkinson, M. (2013). The minimal preprocessing pipelines for the Human Connectome Project. *NeuroImage*, *80*, 105–124. https://doi.org/10.1016/j.neuroimage.2013.04.127

Marcus, D. S., Harms, M. P., Snyder, A. Z., Jenkinson, M., Wilson, J. A., Glasser, M. F., Barch, D. M., Archie, K. A., Burgess, G. C., Ramaratnam, M., Hodge, M., Horton, W., Herrick, R., Olsen, T., McKay, M., House, M., Hileman, M., Reid, E., Harwell, J., … Van Essen, D. C. (2013). Human Connectome Project informatics: Quality control, database services, and data visualization. *NeuroImage*, *80*, 202–219. https://doi.org/10.1016/j.neuroimage.2013.05.077

Van Essen, D. C., Smith, S. M., Barch, D. M., Behrens, T. E. J., Yacoub, E., & Ugurbil, K. (2013). The WU-Minn Human Connectome Project: An overview. *NeuroImage*, *80*, 62–79. https://doi.org/10.1016/j.neuroimage.2013.05.041
